# Performance of mNGS in bronchoalveolar lavage fluid for the diagnosis of invasive pulmonary aspergillosis in non-neutropenic patients

**DOI:** 10.3389/fcimb.2023.1271853

**Published:** 2023-10-31

**Authors:** Ning Zhu, Daibing Zhou, Wanfeng Xiong, Xiujuan Zhang, Shengqing Li

**Affiliations:** Department of Pulmonary and Critical Care Medicine, Huashan Hospital, Fudan University, Shanghai, China

**Keywords:** invasive pulmonary aspergillosis, traditional tests, metagenomic next-generation sequencing, diagnosis, bronchoalveolar lavage fluid

## Abstract

The diagnosis of invasive pulmonary aspergillosis (IPA) diseases in non-neutropenic patients remains challenging. It is essential to develop optimal non-invasive or minimally invasive detection methods for the rapid and reliable diagnosis of IPA. Metagenomic next-generation sequencing (mNGS) in bronchoalveolar lavage fluid (BALF) can be a valuable tool for identifying the microorganism. Our study aims to evaluate the performance of mNGS in BALF in suspected IPA patients and compare it with other detection tests, including serum/BALF galactomannan antigen (GM) and traditional microbiological tests (BALF fungal culture and smear and lung biopsy histopathology). Ninety-four patients with suspicion of IPA were finally enrolled in our study. Thirty-nine patients were diagnosed with IPA, and 55 patients were non-IPA. There was significance between the IPA and non-IPA groups, such as BALF GM (*P* < 0.001), history of glucocorticoid use (*P* = 0.004), and pulmonary comorbidities (*P* = 0.002), as well as no significance of the other demographic data including age, sex, BMI, history of cigarette, blood GM assay, T-SPOT.TB, and NEUT#/LYMPH#. The sensitivity of the BALF mNGS was 92.31%, which was higher than that of the traditional tests or the GM assays. The specificity of BALF mNGS was 92.73%, which was relatively similar to that of the traditional tests. The AUC of BALF mNGS was 0.925, which presented an excellent performance compared with other traditional tests or GM assays. Our study demonstrated the important role of BALF detection by the mNGS platform for pathogen identification in IPA patients with non-neutropenic states, which may provide an optimal way to diagnose suspected IPA disease.

## Introduction

Aspergillosis may cause a spectrum of pulmonary diseases depending on the characteristics of the individual host, including invasive pulmonary aspergillosis (IPA), chronic pulmonary aspergillosis, and allergic bronchopulmonary aspergillosis (Gao and Soubani, 2019). Approximately 250,000 cases of invasive aspergillosis occur annually with a higher mortality (Cornely et al., 2017; Hoenigl et al., 2018). IPA mainly occurs in neutropenic patients and usually increases severe infection. Due to the extensive use of antibiotics, corticosteroids, and immunosuppressants, the prevalence of IPA in patients with non-neutropenia and underlying respiratory disorders, including COPD and interstitial lung disease, has increased in recent years (Dobias et al., 2018; Dai et al., 2021). Thus, the early diagnosis and timely treatment of IPA is a unique challenge.

Although the gold diagnostic standard of IPA is the positive culture of *Aspergillus* spp. from the biopsy species (Kousha et al., 2011), the sensitivity of the fungal smear and the lag of the fungal culture are limited for early pulmonary aspergillosis diagnosis. Meanwhile, patients with suspected IPA often have a pulmonary structural disease, severe thrombocytopenia, and severe clinical conditions. The risk of histopathological examination through CT-guided percutaneous or bronchoscopy biopsy is high or intolerable. Serum and bronchoalveolar lavage fluid (BALF) galactomannan antigen (GM) tests are recognized as a biomarker of *Aspergillus*, widely applied in aspergillosis diagnosis. The GM test is easily affected by antifungal treatment and neutrophil count, leading to a false negative. Therefore, it is essential to develop optimal non-invasive or minimally invasive detection markers, allowing the rapid and reliable diagnosis of IPA.

Metagenomic next-generation sequencing (mNGS) is a new technology for detecting microorganisms that can independently sequence thousands to billions of DNA fragments simultaneously. Compared with traditional tests, mNGS is fast and accurate and has high throughput. mNGS can detect pathological organisms from specimens such as BALF (Li et al., 2020; Zhu et al., 2021), cavity effusion, cerebrospinal fluid, urine, and blood (Ai et al., 2018; Xing et al., 2020). mNGS has been performed in the microbial detection of infectious diseases, including pneumonia (Schlaberg et al., 2017; Huang et al., 2020), bloodstream infection (Blauwkamp et al., 2019), meningitis, and encephalitis (Wilson et al., 2019; Xing et al., 2020; Ramchandar et al., 2021). The application of mNGS shows immense advantages over traditional detection in identifying pathogens (Zinter et al., 2019; Gu et al., 2021; Zhu et al., 2021). However, there are few studies on the diagnostic value of mNGS in non-neutropenia patients with IPA. In this study, we explored the diagnostic accuracy of mNGS in BALF to diagnose suspected IPA in non-neutropenic patients.

## Materials and methods

### Subject

All patients with suspected pulmonary aspergillosis at Huashan Hospital, Fudan University were enrolled and retrospectively investigated from 1 January 2018 to 30 August 2021. The Ethics Committee of Huashan Hospital, Fudan University approved this study, and informed written consent was obtained from each patient’s guardian. The inclusion criteria of suspected IPA were as follows (at least one): a) immunocompromised patients, b) abnormal chest radiographic images suggestive of pulmonary aspergillosis, c) identification of *Aspergillus* genera or species in sputum culture or smear-positive, and d) positive GM test in serum or BALF. All patients were diagnosed and excluded from invasive pulmonary aspergillosis based on the European Organization for the Treatment of Cancer/Mycoses Study Group (EORTC/MSG) guideline (Donnelly et al., 2020). Patients were recognized to have a non-IPA disease if the lesion did not absorb following a course of regular antifungals for 3 months. Patients were excluded if clinical data or informed written consent could not be obtained.

### BALF and lung biopsy specimen

All patients underwent bronchoscopy or lung puncture biopsy, and samples collected from the lesion sites were sent to pathology and culture, respectively. Bronchoscopic alveolar lavage was performed on each patient. If tolerable, histological biopsy in the lung lesions was performed through bronchoscopy or CT-guided percutaneous lung biopsy. Each specimen was divided into two parts for mNGS analysis and conventional tests. All enrolled patients signed an informed consent before undergoing bronchoscopy tests or lung punctures. BALF was obtained through bronchoscopy from the lung lesions according to the standard procedure. Briefly, 60–100 ml saline was injected into the segmental bronchus and withdrawn after a brief wash. An average of 20 ml of BALF samples was divided into two parts and then separately sent to the BGI Genomics (Shenzhen, China) for mNGS analysis and conventional microbiological tests like pathogenic smear and culture. Similarly, lung biopsy specimens were obtained by CT-guided percutaneous fine needle aspiration lung biopsy and were tested by pathology and smear/culture tests. The fungal smear and culture of either BALF or lung biopsy were classified as conventional tests as well as fungal histopathology. If one of them was positive, the patient was considered positive for fungal infection, and no positive results were deemed to be negative.

### Metagenomic next-generation sequencing and bioinformatics analyses

The sampling and detection process is shown in **Figure S1**. According to the manufacturer’s instructions, DNA was extracted from all samples using a QIAamp^®^ UCP Pathogen DNA Kit (Qiagen, Germantown, MD, USA) following the manufacturer’s instructions. Human DNA was removed using Benzonase (Qiagen, Germantown, MD, USA) and Tween 20 (Sigma, St. Louis, MO, USA) (Amar et al., 2021). Total RNA was extracted with a QIAamp^®^ Viral RNA Kit (Qiagen, Germantown, MD, USA), and ribosomal RNA was removed by a Ribo-Zero rRNA Removal Kit (Illumina, San Diego, CA, USA). cDNA was generated using reverse transcriptase and dNTPs (Thermo Fisher, MA, USA). Libraries of DNA and cDNA samples were constructed using a Nextera XT DNA Library Prep Kit (Illumina, San Diego, CA, USA) (Miller et al., 2019). The quality of the library was assessed by a Qubit dsDNA HS Assay kit followed by a High Sensitivity DNA kit (Agilent, Santa Clara, CA, USA) on an Agilent 2100 Bioanalyzer. Library pools were then loaded onto an Illumina NextSeq CN500 sequencer for 75 cycles of single-end sequencing to generate approximately 20 million reads for each library.

Trimmomatic was used to remove low-quality reads, adapter contamination, duplicate reads, and those shorter than 50 bp. Kcomplexity removed low-complexity reads with default parameters. Human sequence data were identified and excluded by mapping to a human reference genome (hg38) using the Burrows–Wheeler Aligner software. We designed a set of criteria similar to the National Center for Biotechnology Information (NCBI) criteria for selecting the representative assembly for microorganisms (bacteria, viruses, fungi, protozoa, and other multicellular eukaryotic pathogens) from the NCBI Nucleotide and Genome databases (https://www.ncbi.nlm.nih.gov/assembly/help/anomnotrefseq/, Accessed March 2021). Pathogen lists were selected according to three references: 1) Johns Hopkins ABX Guide (https://www.hopkinsguides.com/hopkins/index/Johns_Hopkins_ABX_Guide/Pathogens), 2) Manual of Clinical Microbiology, and 3) clinical case reports or research articles published in current peer-reviewed journals (Fiorini et al., 2017). The final database consisted of approximately 13,000 genomes. Microbial reads were aligned to the database with SNAP v1.0beta.18. Virus-positive detection results (DNA or RNA viruses) were defined as covering three or more non-overlapping regions on the genome. A positive detection was reported for a given species or genus if the reads per million (RPM) ratio or RPM-r was ≥5, where the RPM-r was defined as the RPM_sample_/RPM_NC_ (i.e., the RPM corresponding to a given species or genus in the clinical sample divided by the RPM in the NC/negative control) (Miller et al., 2019). In addition, to minimize cross-species misalignments among closely related microorganisms, we penalized (reduced) the RPM of microorganisms sharing a genus or family designation if the species or genus appeared in non-template controls. A penalty of 5% was used for the species (Gu et al., 2021).

### GM antigen assay

The *Aspergillus* GM antigen assay in BALF and peripheral blood was quantified using a Platelia *Aspergillus* double sandwich enzyme-linked immunosorbent assay kit (Bio-Rad, USA). The optical density index (ODI) of GM was calculated. ODI in serum or BALF refers to the sample/standard value according to the manufacturer’s protocol. The ODI of GM <0.5 is negative, and ≥0.5 is regarded as positive. In this study, we defined the ODI ≥0.717 as the optimal cutoff value.

### Statistical analysis

The enrolled patients were divided into IPA and non-IPA infection groups according to the final clinical diagnosis and microbiological etiology. The Student’s *t*-test and chi-squared test or Fisher’s exact test were used to identify group differences. Continuous variables were reported as mean and standard deviation (SD) and calculated using the Student’s *t*-test. To determine the sensitivity, specificity, positive predictive value (PPV), and negative predictive value (NPV), 2 × 2 contingency tables were derived. Comparison of sensitivity, specificity, AUC, and ROC curve analysis between groups was performed by the MedCalc software (MedCalc Software Ltd: Ostend, Belgium). The other statistical analyses were performed using SPSS 22.0 (IBM Corp., Armonk, NY, USA). All statistics have been reported as absolute values with their 95% confidence interval (95% CI). A two-sided *P*-value less than 0.05 was considered to be statistically significant.

## Results

### Patient demographics

**Figure 1:**
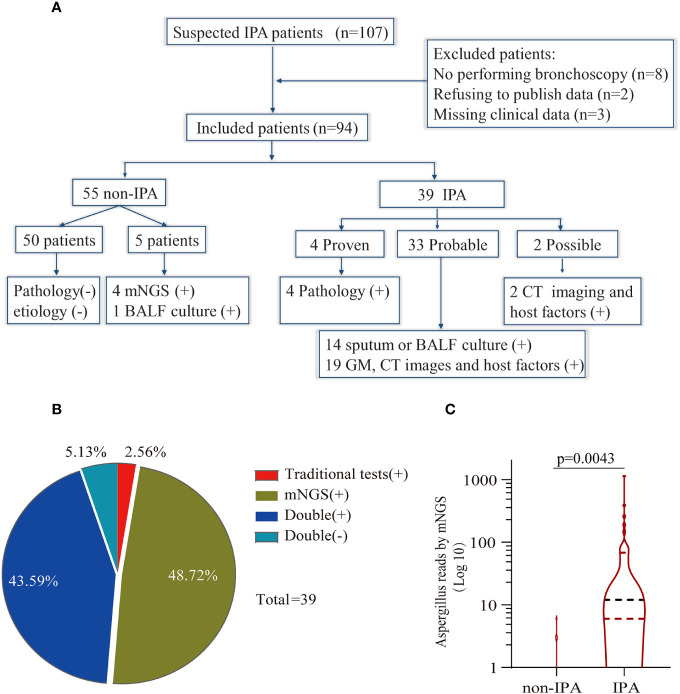
Study workflow and data analysis. **(A)** Schedule of all enrolled patients: 107 suspected patients were initially enrolled in this retrospective study, and 94 patients were finally included in the analysis. Fifty-five patients were diagnosed as non-invasive pulmonary aspergillosis (IPA), and 39 patients were diagnosed as IPA. **(B)** Concordance between metagenomic next-generation sequencing (mNGS) and traditional detection methods in the diagnosis of IPA. The pie chart shows the positive rate of mNGS and traditional detection methods. Among the 39 IPA patients, 43.59% were positive for both mNGS and traditional tests, and 5.13% were negative for mNGS and traditional tests. Meanwhile, only mNGS was positive in 48.72% of patients, and 2.56% were positive by traditional test methods. **(C)** Comparison of the number of unique reads between the IPA group and the non-IPA group. The number of unique reads was significantly higher in the IPA group than that in the non-IPA group.

One hundred and seven patients with suspected IPA were initially enrolled in our study from 1 January 2018 to 30 August 2021. Eight patients were excluded for refusing to undergo bronchoscopy to obtain BALF, two for refusing to publish their clinical data, and three for missing clinical data. Ninety-four patients were finally enrolled in this study. Thirty-nine patients were diagnosed with IPA, consisting of 4 proven patients, 33 probable patients, and 2 possible patients. The classification of *Aspergillus* sp. in IPA patients included 29 *Aspergillus fumigatus*, 4 *Aspergillus flavus*, 1 *Aspergillus terricola*, 1 *Aspergillus nidulans*, and 4 unclassified-type *Aspergillus* spp. Fifty-five patients were definitely diagnosed with non-IPA (**Figure 1A**).

**Figure 2:**
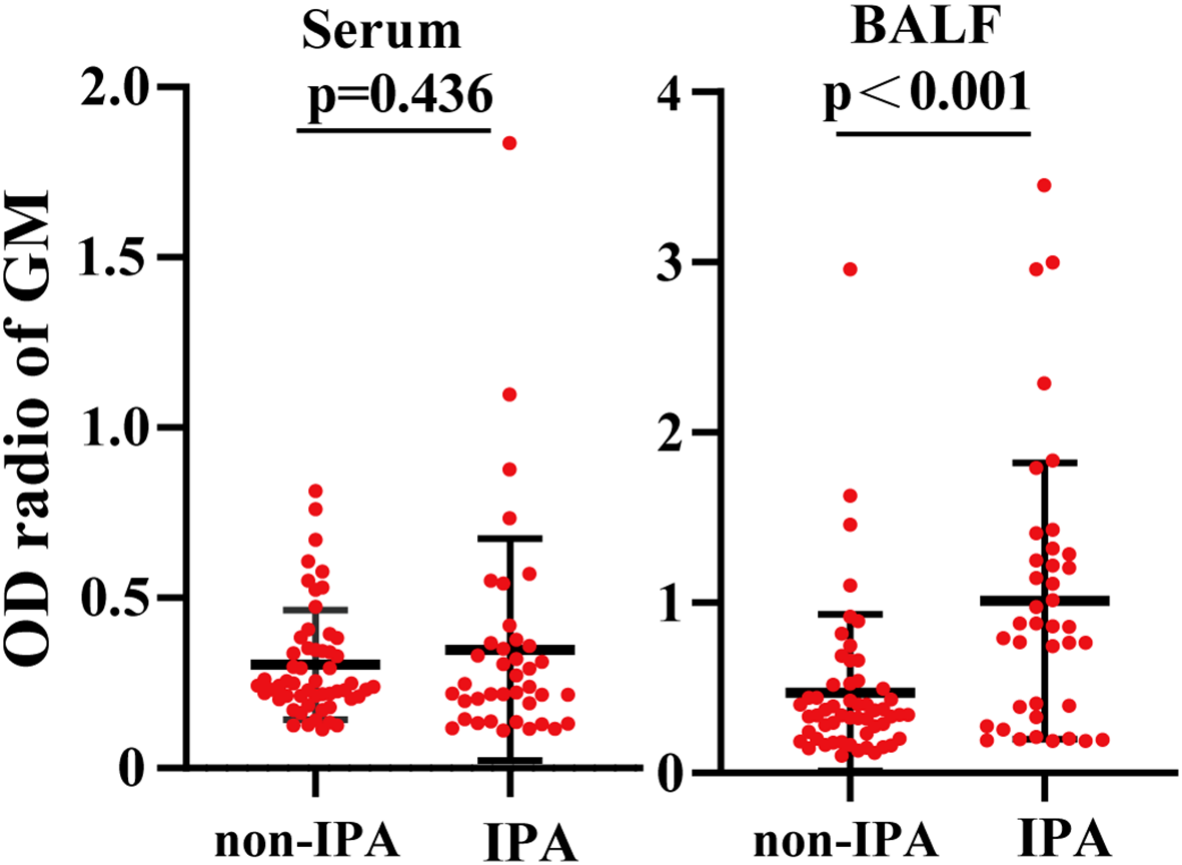
Comparison of the galactomannan antigen (GM) levels between the IPA group and the non-IPA group. Compared with the BALF GM levels in the non-IPA group, the BALF GM levels were significantly higher. However, the two groups had no significant difference in serum GM levels.

The primary characteristics of all the eligible patients are presented in **Table 1**. There was no significant difference between the IPA and non-IPA groups in most of the demographic data [including gender, age, BMI, serum GM, T-SPOT.TB assay, and the ratio of neutrophil (NEUT#)/lymphocyte (LYMPH#)]. However, we observed that patients with a history of cigarette smoking, glucocorticoid usage, and underlying pulmonary comorbidities were preferential to suffer from IPA (*P* < 0.01). Patients who presented with underlying pulmonary diseases (23/33), especially COPD, pulmonary tuberculosis, lung cancer, etc., were more susceptible to IPA (**Table S1**). Among the 39 IPA patients, 43.59% of patients were positive both in the mNGS and traditional tests, 5.13% were negative both in the mNGS and traditional tests, 48.72% were positive only in the mNGS, and 2.56% were positive only in the traditional test (**Figure 1B**). We further analyzed the number of unique reads in the mNGS between the IPA and non-IPA groups. The number of unique reads was significantly higher in the IPA group than that in the non-IPA group (*P* = 0.0043) (**Figure 1C**). Compared with the non-IPA group, we found no significance of GM in the serum between the IPA and non-IPA groups (*P* = 0.436). However, BALF GM was significantly higher in the IPA group (1.01 ± 0.81 vs. 0.47 ± 0.46; *P*<0.001), which indicated that the BALF GM assay might be an optional diagnostic assay for IPA (**Figure 2**). Although imaging findings were not prominent, including multiple nodules, patchy shadows, emphysema, and bullae caused by to the non-IPA infection, cavities and bronchiectasis were most prevalent in IPA infection based on a retrospective review of radiological images (**Table S2**).

**Table 1:** Baseline characteristics of IPA and non-IPA patients.

Characteristic	*N*	IPA	Non-IPA	*P*-value
**No. of patients**	94	39	55	
**Age** (years)		57.46 ± 15.52	56.47 ± 15.00	0.757
**Sex,** *n* (%)				0.207
Male	53	19 (48.72)	34 (61.82)	
Female	41	20 (51.28)	21 (38.18)	
**BMI**		22.42 ± 3.50	22.77 ± 2.99	0.597
Smoking history (≥5 years)				
**Male**	53			0.039
Yes		14	17	
No		5	17	
**Female**	41			>0.99
Yes		1	1	
No		19	20	
**Serum GM**		0.35 ± 0.33	0.30 ± 0.16	0.436
**BALF GM**		1.01 ± 0.81	0.47 ± 0.46	<0.001
**T-SPOT.TB**				0.86
Positive	25	10	15	
Negative	69	29	40	
**Glucocorticoid usage** (≥3 weeks)				0.004
Yes	20	14	6	
No	74	25	49	
**Pulmonary disease**				0.002
Yes	47	27	20	
No	47	12	35	
**NEUT#/LYMPH#** (range 0.9~3.1)				0.864
≥3.1	40	17	23	
≥0.9, <3.1	54	22	32	

a, Independent-samples t-test; b, chi-squared test. BMI, Body Mass Index; NEUT, Neutrophils; LYMPH, Lymphocyte.

### Comparison of sensitivity and specificity in serum/BALF GM, traditional test, and BALF mNGS

**Figure 3:**
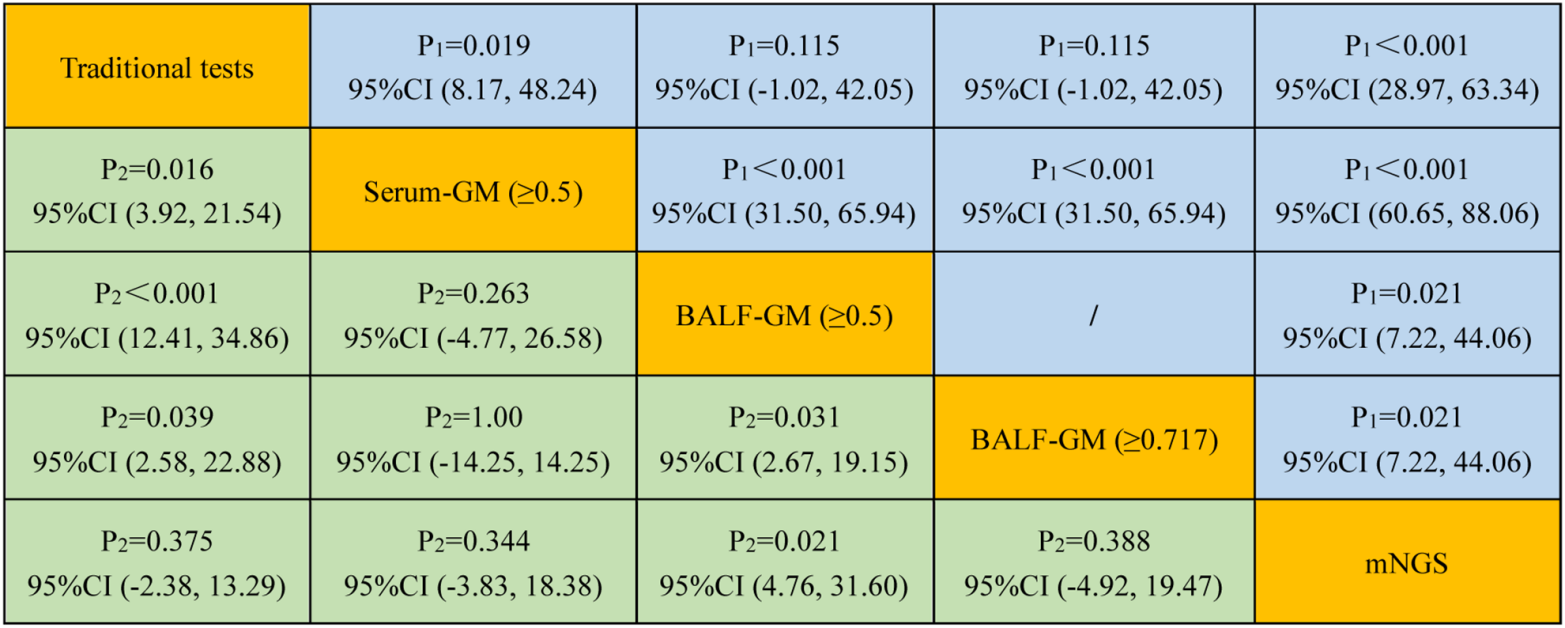
Comparison of the sensitivity and specificity among different diagnostic methods for suspected IPA. P1 and P2 represent the comparative analysis of sensitivity and specificity between two special groups, respectively.

An optical density (OD) index of ≥0.5 in the serum GM assay (serum GM 0.5) is recommended for the suspected IPA diagnosis. As shown in **Table 2**, when the OD ratio cutoff value of the GM test in the serum was set to 0.5, the sensitivity, specificity, PPV, and NPV were 17.95% (95% CI: 7.50, 33.50), 85.45% (95% CI: 73.30, 93.50), 46.67% (95% CI: 25.70, 68.90), and 59.49% (95% CI: 55.00, 63.80), respectively. Then, we evaluated the diagnostic efficiency of the traditional tests, and the sensitivity, specificity, PPV, and NPV were 46.15% (95% CI: 30.10, 62.80), 98.18% (95% CI: 90.30, 100.0), 94.70% (95% CI: 71.50, 99.20), 72.00% (95% CI: 65.70, 77.50), respectively. Furthermore, it showed higher sensitivity and specificity when compared with those of serum GM [*P* = 0.019, 95% CI (8.17, 48.24); *P* = 0.016, 95% CI (3.92, 21.54)] (**Figure 3**).

**Table 2:** Diagnostic performance of traditional tests, serum/BALF GM, and mNGS in the diagnosis of suspected IPA.

	IPA	Non-IPA	Sensitivity% (95% CI)	Specificity% (95% CI)	PPV% (95% CI)	NPV% (95% CI)	Youden index	*P*-value
**Serum GM (≥0.50)**			17.95 (7.50, 33.50)	85.45 (73.30, 93.50)	46.67 (25.70, 68.90)	59.49 (55.0, 63.80)	0.034	0.665
Yes	7	8						
No	32	47						
**Traditional tests**			46.15 (30.10, 62.80)	98.18 (90.30, 100.0)	94.70 (71.50, 99.20)	72.0 (65.70, 77.50)	0.443	<0.001
Yes	18	1						
No	21	54						
**BALF GM (≥0.50)**			66.67 (49.80, 80.90)	74.55 (61.0, 85.30)	65.0 (52.90, 75.50)	75.90 (66.30, 83.50)	0.412	<0.001
Yes	26	14						
No	13	41						
**BALF GM (≥0.717)**			66.67 (49.80, 80.90)	85.45 (73.30, 93.50)	76.50 (62.30, 86.50)	78.30 (69.60, 85.10)	0.521	<0.001
Yes	26	8						
No	13	47						
**BALF mNGS**			92.31 (79.10, 98.40)	92.73 (82.40, 98.00)	90.0 (77.70, 95.90)	94.40 (85.10, 98.10)	0.850	<0.001
Yes	36	4						
No	3	51						

Because the concentration of GM in BALF may be higher than that in the serum, we further evaluated the diagnostic value of BALF GM for IPA. When the threshold value of the GM assay was 0.5, the sensitivity, specificity, PPV, and NPV of BALF GM were 66.67% (95% CI: 49.80, 80.90), 74.55% (95% CI: 61.0, 85.30), 65.0% (95% CI: 52.90, 75.50), and 75.90% (95% CI: 66.30, 83.50), respectively (**Table 2**). These results showed that the BALF GM assay might have a higher diagnostic efficiency of IPA than the serum GM or traditional tests.

**Figure 4:**
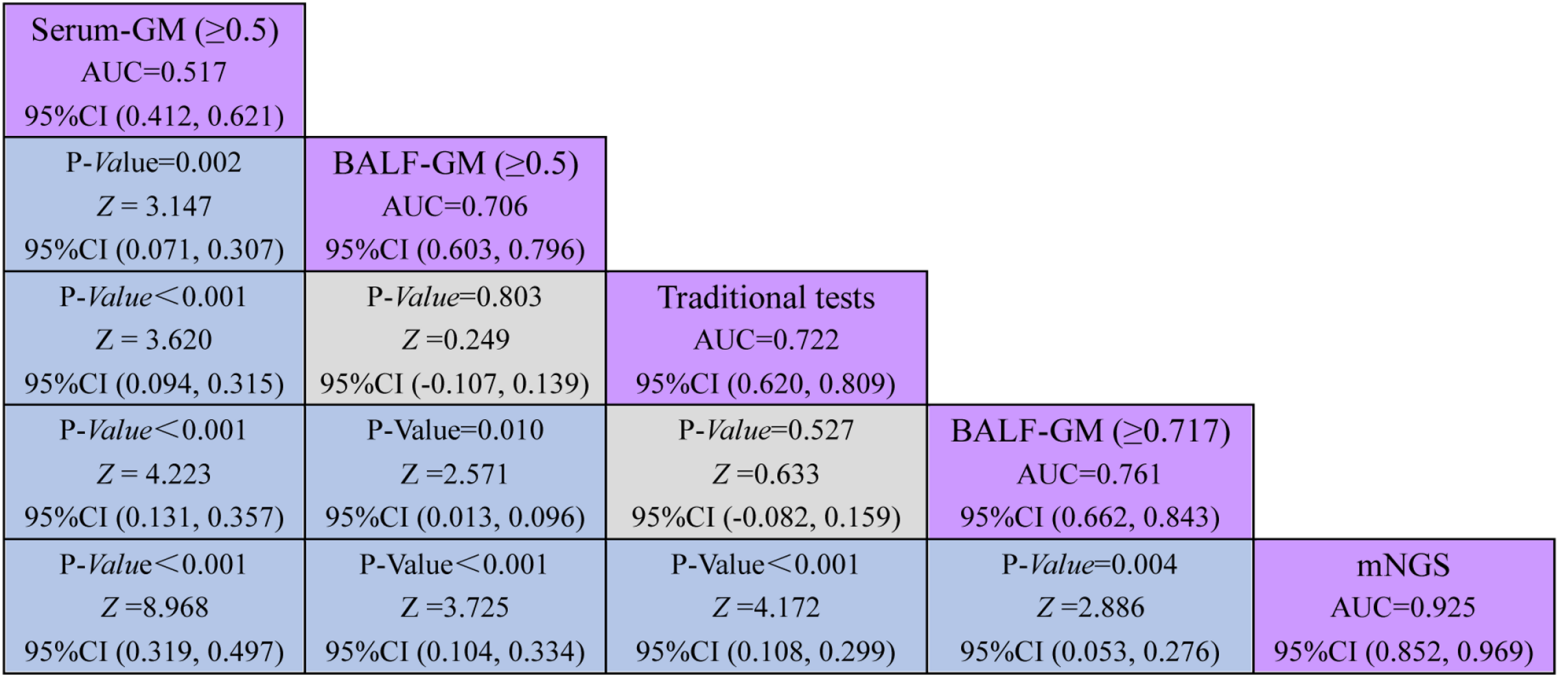
Comparative analysis of different assays by ROC. ROC curves evaluated the diagnostic efficiency of these several detection assays. The AUC of BALF mNGS was 0.925 (95% CI: 0.852, 0.969), significantly higher than the other assays.

Our study found that the optimal cutoff value in BALF GM obtained was 0.717 by ROC (**Figure 4**). When the cutoff value reached 0.717, the sensitivity, specificity, PPV, and NPV were 66.67% (95% CI: 49.80, 80.90), 85.45% (95% CI: 73.30, 93.50), 76.50% (95% CI: 62.30, 86.50), and 78.30% (95% CI: 69.60, 85.10), respectively (**Table 2**). As presented in **Figure 3**, although there was no significant difference between the sensitivity of the two BALF GM levels (*P* = 1.00), the specificity of BALF GM (≥0.717) significantly increased [*P* = 0.031, 95% CI (2.67, 19.15)]. It suggested that when the cutoff value increased from 0.5 to 0.717, BALF GM assays could significantly reduce the false-positive rate. Similarly, the sensitivity of BALF GM (≥0.717) was similar to that of traditional tests [*P* = 0.115, 95% CI (−1.02, 42.05)], but the specificity of BALF GM (≥0.717) was significantly higher [*P* = 0.039, 95% CI (2.58, 22.88)].

### Performance of mNGS in BALF outperforming the traditional test and GM assay

**Figure 5:**
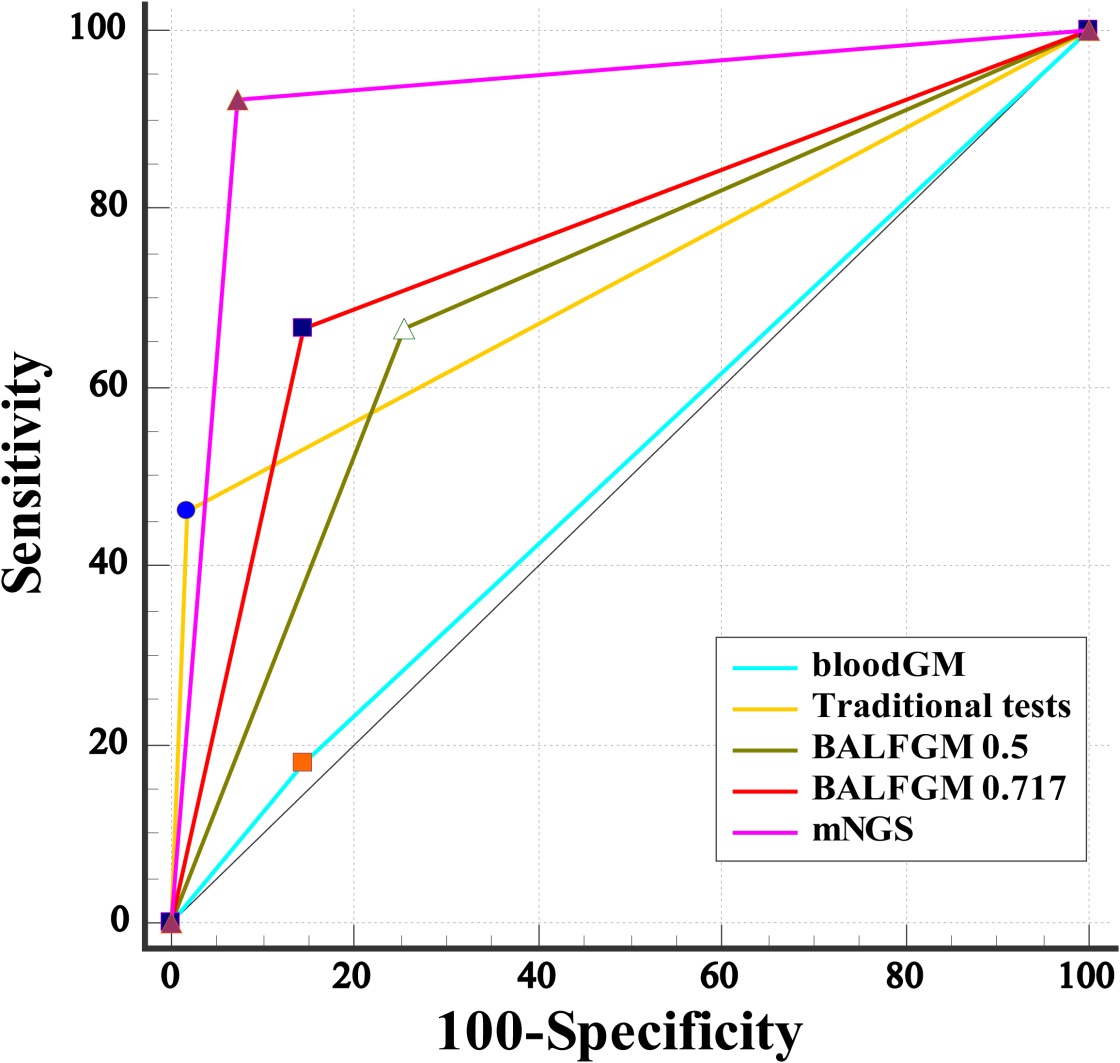
Comparison of different diagnostic methods through the ROC curve.

As provided in **Table 2**, the sensitivity, specificity, PPV, and NPV of BALF mNGS were 92.31% (95% CI: 79.10, 98.40), 92.73% (95% CI: 82.40, 98.00), 90.0% (95% CI: 77.70, 95.90), and 94.40% (95% CI: 85.10, 98.10). The ROC curves also evaluated the diagnostic accuracy of these several detection assays. The ROC curves of the GM assay, traditional test, and mNGS are shown in **Figure 3**. When the cutoff value was set to 0.5, the AUC of serum GM was 0.517, with a 95% confidence index (0.412, 0.621). The AUC of BALF GM (≥0.5) was 0.706 (95% CI: 0.603, 0.796), which was much higher than the AUC of serum GM. Meanwhile, it was pretty similar to the AUC of the traditional test (0.722, 95% CI: 0.620, 0.809), without significant difference (*P* = 0.803). When the cutoff value reached up to 0.717, the ROC curves showed the superiority of the diagnostic accuracy of BALF GM (≥0.717) to be significantly increased (0.761, 95% CI: 0.662, 0.843). Furthermore, the AUC of BALF mNGS was 0.925 (95% CI: 0.852, 0.969), significantly higher than the other assays. It indicated that the diagnostic accuracy of BALF mNGS for suspected IPA was significantly higher than the other diagnostic assays (**Figure 5**).

## Discussion

Early recognition and timely diagnosis are crucial to improve the clinical outcome of patients with IPA. The application of non-invasive biochemical markers, including serum/BAL fungal cell wall antigens, GM, β-D glucan, and *Aspergillus* polymerase chain reaction (PCR), could provide options for the identification of pulmonary *Aspergillus* infection (Boch et al., 2018; Hage et al., 2019; Haydour et al., 2019; Bassetti et al., 2020). Previous studies found that the BALF GM test is more efficient for IPA than the serum GM test (Meersseman et al., 2008; Jenks et al., 2020; Lai et al., 2020). A study revealed that the sensitivity and specificity of the BALF GM test were only 88% and 87%, respectively, when the cutoff value of the GM test was 0.5 in patients with non-granulocyte deficiency. In comparison, the sensitivity of the serum GM test was only 42% (Meersseman et al., 2008). In our study, we revealed that the sensitivity and specificity of BALF GM (≥0.717) could reach up to 66.67% and 85.45%, respectively, and the AUC is 0.761, which was quite in accordance with other previous studies (Meersseman et al., 2008; Zhou et al., 2017; Lai et al., 2020). However, antifungal treatment and neutrophil counts easily affect the GM test. It might cause false-negative results, especially in non-neutropenic patients. So, it could not significantly improve the diagnostic accuracy for aspergillosis.

A positive culture of *Aspergillus* in biopsy was recommended for the final diagnosis of pulmonary *Aspergillus* infection (El-Baba et al., 2020). However, relying on culture to diagnose a fungal infection might lead to a poor outcome due to its limited sensitivity and false positivity. BALF and lung biopsy are mainly examined in suspected IPA infection (Nikbakhsh et al., 2015; Li et al., 2018; Khan and Aladab, 2020; Luo et al., 2020; Yang et al., 2021). In addition, patients with suspected IPA often have lung structural damage, coagulation disorders, and severe clinical manifestation. Lung biopsy easily leads to bleeding or pneumothorax, which is likely life-threatening. BALF was the optimal specimen for detecting *Aspergillus* spp. in our study.

mNGS has been widely used in detecting microorganisms for infectious diseases. Unlike traditional tests, mNGS allows thousands to billions of DNA fragments to be independently sequenced simultaneously and is not influenced by genomic mutations or diversity (Filkins et al., 2020). A study indicated that mNGS could identify at least one microbial species in nearly 89% of cases of pulmonary infection and present positive pathogenic results in 94.49% of specimens in pulmonary infection patients who were negative by traditional tests (Huang et al., 2020). However, for 106 patients with suspected pulmonary fungal infection, lung biopsy mNGS and BALF mNGS presented no significance in sensitivity and specificity (Yang et al., 2021). BALF mNGS is still preferred to lung biopsy mNGS by comprehensive risk assessment. mNGS provided specific sequencing copies of all microorganisms, including bacterium, fungi, tuberculosis, *Mycoplasma*, chlamydia, and DNA viruses (Gu et al., 2019; Chen et al., 2020; Liu D. et al., 2021; Zhu et al., 2021), and identified additional pathogens of hematologic patients with sepsis (Liu WD. et al., 2021). It has a potential role in non-reported and emerging pathogens (Ngoi et al., 2016; Casto et al., 2019; Carbo et al., 2021). An mNGS study reported a typical pathogen infection, which still identified no noticeable pathogens by traditional methods during 2 years (Thoendel et al., 2017). Our previous study showed that BALF- or lung biopsy-based mNGS could improve TB infection’s diagnostic accuracy for sputum-scarce or smear-negative patients (Zhu et al., 2021). mNGS detection was recommended as follows (Filkins et al., 2020): i) patients with suspected unculturable and atypical infection, ii) acquired antibiotic exposure or persistent immunosuppression, iii) negative results by conventional methods, and iv) patients with severe infectious diseases or septic shock. Interestingly, the platform of mNGS detection of lung biopsy could be applied to examine cancer and pathogens based on Illumina sequencing for patients with abnormal radiological images, and the pipeline provided a neophyte perspective toward the mNGS method (Guo et al., 2021).

The application of mNGS in immunosuppressive patients with severe community-acquired pneumonia and transplant infection could be advantageous in detecting mixed pathogenic infections and guiding decisions on antibiotic prescription (Young et al., 2019; Sun et al., 2021). However, the value of mNGS in the diagnosis of IPA is still less explored, especially in non-neutropenic patients. In our study, we revealed that the sensitivity of BALF mNGS was 92.31% for diagnosing IPA in non-neutropenic patients, significantly higher than other detection assays. The experimental principle of mNGS could easily explain it. The specificity of BALF mNGS was equivalent to conventional culture. The AUC of BALF mNGS was 0.925. These results indicated that BALF mNGS could have higher diagnostic accuracy than those of several other assays.

The major advantage of mNGS is its wide detection range and precise and timely microbial diagnoses (Langelier et al., 2018; Wilson et al., 2019), presenting a higher diagnostic sensitivity and specificity (Li et al., 2020). Despite the encouraging results of our study, there are still some limitations in our study. Firstly, DNA extraction efficiency is critical to mNGS results. Due to the thick cell walls of fungi and intracellular bacteria, wall-breaking treatment was applied in the DNA extraction process. Still, inadequate wall-breaking treatment might cause false-negative results. Meanwhile, bioinformatics analysis and interpretation of sequencing results also affect all aspects of the final results of mNGS. Secondly, the accuracy of mNGS is susceptible to nucleic acid contamination in the background microbiome and reaction kit. It was difficult to distinguish infection from colonization and outside nucleic acid sources. Moreover, it is difficult to determine the optimal threshold for pathogen identification. Thirdly, our sample size was relatively limited, which would be attributed to the bias. Thus, it is necessary to make a further study. Finally, mNGS is not commonly performed due to high cost and time-consuming data interpretation, especially in developing countries. The average fee of mNGS is approximately 3,000 yuan (RMB) per sample, which was still a substantial economic burden for most patients.

In summary, our study demonstrated the critical role of BALF detection by the mNGS platform for pathogen identification in IPA patients with non-neutropenic states. It may provide an optimal way to diagnose the suspected IPA disease, especially for those hard to acquire enough candidate samples and present negative results by traditional methods.

## Data availability statement

The data presented in the study are deposited in the figshare dataset repository, accession number: https://figshare.com/s/0a8f5bc3b2caed19da6e.

## Ethics statement

The studies involving humans were approved by Huashan Hospital, Fudan University. The studies were conducted in accordance with the local legislation and institutional requirements. The participants provided their written informed consent to participate in this study.

## Author contributions

NZ: Conceptualization, Data curation, Writing – original draft. DZ: Conceptualization, Formal Analysis, Writing – original draft. WX: Data curation, Methodology, Writing – review & editing. XZ: Methodology, Supervision, Writing – original draft. SL: Conceptualization, Supervision, Writing – review & editing.

## Supplementary material

Supplementary Figure 1 Schematic of mNGS analysis workflow for bronchoalveolar lavage fluid or lung tissue.
