# Supplementary material for: Performance of mNGS in bronchoalveolar lavage fluid for the diagnosis of invasive pulmonary aspergillosis in non-neutropenic patients
Source: Front Cell Infect Microbiol. 2023 Oct 31;13:1271853. doi: 10.3389/fcimb.2023.1271853 (PMC10644336; doi:10.3389/fcimb.2023.1271853)
Supplement: Supplementary file 1 [file Table_1.docx]

**Supplementary Table 1**. Composition analysis of IPA patients.

| Pulmonary Complications | IPA  (N=39) | Non-IPA  (N=55) |
| --- | --- | --- |
| **Pulmonary diseases** |  |  |
| Bronchiectasis | 13 | 5 |
| Asthma | 2 | 0 |
| Interstitial pneumonia | 2 | 2 |
| Lung cancer | 5 | 2 |
| Tuberculosis (pleurisy) | 7 | 1 |
| emphysema, chronic bronchitis | 13 | 4 |
| COP | 0 | 1 |
| SAHS | 0 | 1 |
| PTE | 1 | 0 |
| **Rheumatic immune disease** |  |  |
| Sjogren Syndrome | 1 | 0 |
| Dermatomyositis/poly myositis | 1 | 0 |
| **Myelodysplastic syndromes** | 1 | 0 |
| **Acute Lymphoblastic leukemia** | 1 | 0 |
| **Myasthenia Gravis** | 0 | 1 |
| **Diabetes** | 8 | 10 |
| **Hypertension** | 8 | 15 |
| **Coronary heart disease** | 2 | 1 |
| **History of surgery** | 7 | 4 |
| **Other cancers** |  |  |
| Thymus tumor | 0 | 2 |
| Thyroid tumor | 1 | 1 |
| Laryngeal cancer | 1 | 0 |
| Kidney cancer | 0 | 1 |
| Ovarian cancer | 0 | 1 |
