# Supplementary material for: Performance of mNGS in bronchoalveolar lavage fluid for the diagnosis of invasive pulmonary aspergillosis in non-neutropenic patients
Source: Front Cell Infect Microbiol. 2023 Oct 31;13:1271853. doi: 10.3389/fcimb.2023.1271853 (PMC10644336; doi:10.3389/fcimb.2023.1271853)
Supplement: Supplementary file 2 [file Table_2.docx]

**Supplementary Table 2**. Characteristics of pulmonary CT images in patients.

| Features of CT images | IPA  (N=39) | *Non-IPA*  (N=55) |
| --- | --- | --- |
| Cavities | 5 | 1 |
| Multiple nodules | 10 | 27 |
| Bronchiectasis | 13 | 4 |
| Patchy shadows | 23 | 26 |
| Lung mass (≥3cm) | 1 | 2 |
| Emphysema, bullae | 5 | 5 |
| Pleural effusion | 2 | 1 |
| Mediastinal lymphadenopathy | 0 | 2 |
| Interstitial images | 3 | 2 |
