# Supplementary figures and images for: Performance of mNGS in bronchoalveolar lavage fluid for the diagnosis of invasive pulmonary aspergillosis in non-neutropenic patients
Source: Front Cell Infect Microbiol. 2023 Oct 31;13:1271853. doi: 10.3389/fcimb.2023.1271853 (PMC10644336; doi:10.3389/fcimb.2023.1271853)

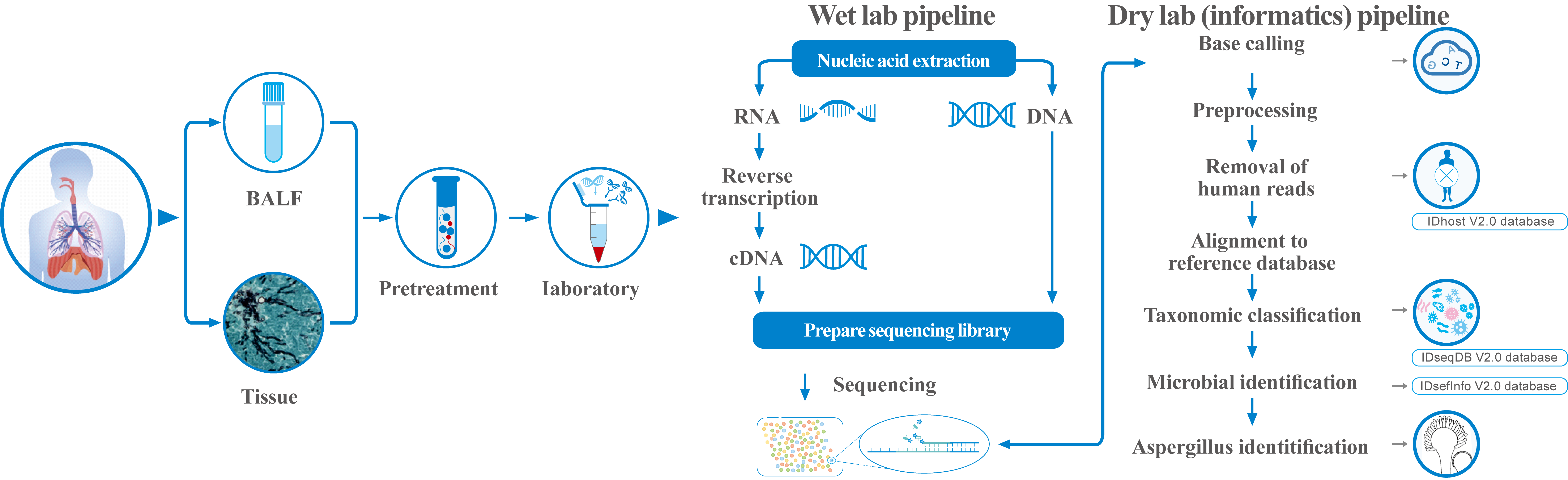

Supplement: Supplementary Figure 1 — Schematic of mNGS analysis workflow for bronchoalveolar lavage fluid or lung tissue. [file Image_1.tif]
